# Predicting judging-perceiving of Myers-Briggs Type Indicator (MBTI) in online social forum

**DOI:** 10.7717/peerj.11382

**Published:** 2021-06-23

**Authors:** En Jun Choong, Kasturi Dewi Varathan

**Affiliations:** Department of Information Systems, Faculty of Computer Science & Information Technology, Universiti Malaya, Kuala Lumpur, Malaysia

**Keywords:** Myers-Briggs Type Indicator, MBTI, Personality Computing, Judging-Perceiving, Light Gradient Boosting, Natural Language Processing

## Abstract

The Myers-Briggs Type Indicator (MBTI) is a well-known personality test that assigns a personality type to a user by using four traits dichotomies. For many years, people have used MBTI as an instrument to develop self-awareness and to guide their personal decisions. Previous researches have good successes in predicting Extraversion-Introversion (E/I), Sensing-Intuition (S/N) and Thinking-Feeling (T/F) dichotomies from textual data but struggled to do so with Judging-Perceiving (J/P) dichotomy. J/P dichotomy in MBTI is a non-separable part of MBTI that have significant inference on human behavior, perception and decision towards their surroundings. It is an assessment on how someone interacts with the world when making decision. This research was set out to evaluate the performance of the individual features and classifiers for J/P dichotomy in personality computing. At the end, data leakage was found in dataset originating from the Personality Forum Café, which was used in recent researches. The results obtained from the previous research on this dataset were suggested to be overly optimistic. Using the same settings, this research managed to outperform previous researches. Five machine learning algorithms were compared, and LightGBM model was recommended for the task of predicting J/P dichotomy in MBTI personality computing.

## Introduction

Two most prevailing personality models are the Big Five Inventory (BFI) and Myers-Briggs Type Indicator (MBTI). Unlike the BFI, which is a trait-based approach, the MBTI assessment is a type-based approach. MBTI assessment model is used in 115 countries with 29 languages available, and it is used by 88 of the Fortune 100 within the past five years (Kerwin, 2018). MBTI is a world-renowned assessment and practitioners have placed far more trust in it than did organization scholar (Lake et al., 2019).

Thanks to the widespread dissemination of free personality assessment models online, many people are sharing their personality on social media. Large scale self-reported personality assessment results had been made available conveniently through the means of datamining on social media platform. This is evident through Plank & Hovy (2015), where a corpus of 1.2 M tweets from 1,500 users that self-identified with an MBTI type were collected from Twitter within a week. Subsequently, a few more datasets on MBTI became available through social media platform, resonating the ease of data collection (Verhoeven, Plank & Daelemans, 2016; Celli & Lepri, 2018; Gjurković & Šnajder, 2018).

MBTI remains largely popular and outperform BFI in specific domains (Yoon & Lim, 2018; Yi, Lee & Jung, 2016). MBTI consists of four pairs of opposing dichotomies, namely: Extraversion-Introversion (E/I), Sensing-Intuition (S/N), Thinking-Feeling (T/F) and Judging-Perceiving (J/P). Accurate inference of users’ personality is substantial to the performance of downstream applications. One such application is personalized advertisement on social media. According to Shanahan, Tran & Taylor (2019), firm spending on social media marketing has more than quadrupled in the past decade; their result further suggests that social media personalization positively impacts consumer brand engagement and brand attachment. Self-reported personality assessments are not common across social media platform and represent a negligible population of the social media users. A more scalable and sustainable way to inference users’ personality is thru linguistic features from users’ interactions on social media.

In recent years, researches such as Li et al. (2018) have successes in prediction particularly E/I, S/N and T/F dichotomies with above 90% accuracy. However, most researches struggled with predicting J/P dichotomy from textual data. Judging-Perceiving (J/P) dichotomy in MBTI is a non-separable part of MBTI that dictates a person lifestyle preference which can have significant predictive power to infer human-behavior in real-world use cases. While the three dichotomies aside from J/P can be known for a person, a complete picture cannot be painted without knowing the J/P dichotomy of the person. The predicament on predicting this dichotomy more than the others had manifested itself consistently thru the poor prediction performance of previous researches data (Li et al., 2018; Lukito et al., 2016; Verhoeven, Plank & Daelemans, 2016; Plank & Hovy, 2015; Wang, 2015). The poor prediction performance on J/P dichotomy can affect one’s personal decision guided by the misrepresented dichotomy class. This can lead to distrust and stall development of MBTI in various real-world application. Since MBTI is not on a continuous scale, but of four opposing dichotomies, a wrong prediction would mean incorrectly predicting the opposing extreme of a type. This would give a conflicting effect. For instance, a wrong prediction in the J/P dichotomy could mean recommending a career which requires high order of structure like an engineer to a “Perceiving” type person who prefer more creativity and flexibility. Thus, it is crucial to be able to predict J/P dichotomy alike other three dichotomies with high confidence.

Researches till date on social media MBTI personality computing have focused on predicting the four personality pairs indifferently. Prediction on J/P dichotomy in past researches have been consistently underperforming the three other dichotomies. This is reflected in recent researches such as Lima & Castro (2019), Yamada, Sasano & Takeda (2019) and Keh & Cheng (2019), etc. A dedicated study on J/P dichotomy is key to tighten the performance gap among the four dichotomies in MBTI prediction. The J/P dichotomy is the crucial piece of puzzle to solve in MBTI prediction, by improving the prediction, a complete picture of the user’s MBTI personality can be inferred. Furthermore, J/P dichotomy alone is enough to infer insightful behaviors (Kostelic, 2019; Pelau, Serban & Chinie, 2018; Yoon & Lim, 2018; Wei et al., 2017).

The objectives of this research are focused on the prediction of the Judging/Perceiving dichotomy of MBTI. In order to do so, the research identified and selected a few linguistic features, subsequently run them through five classifiers, then finally recommend the best combination for this task. The rest of the paper is organized as follows: ‘Related Works’ presents the related works of the study followed by ‘Methodology’ on research methodology. ‘Results & Discussion’ catered for results and discussions. Finally, ‘Conclusion & Future Work’ concludes the research with the summary of the findings and future work.

## Related Works

This section starts by providing a background of the four dichotomies in MBTI. An elaborative search and review of research pertaining to personality computing using online social forum dataset were conducted. As a result of that, personality computing for Judging/Perceiving dichotomy in MBTI were identified as the worst performing dichotomy and as the research gap of this research (Li et al., 2018; Lukito et al., 2016; Verhoeven, Plank & Daelemans, 2016; Plank & Hovy, 2015; Wang, 2015). The importance of Judging/Perceiving dichotomy was emphasized, and methodologies commonly used in this discipline were identified.

### Myers-Briggs types indicator (MBTI)

The MBTI assessment is designed to identify personality preferences (Kerwin, 2018). MBTI find its relevance and effectiveness in the communication and development arena, where one deal with career planning, conflict management, organization team building, self-reflection, etc. (Moyle & Hackston, 2018). The author is aware of the criticism on MBTI in the past few decades, but it has little impact on MBTI’s popularity (Stein & Swan, 2019), and those criticism were well countered (Kerwin, 2018). In fact, recent study by Yoon & Lim (2018) demonstrated that MBTI preferences significantly affect impulsive and compulsive buying in online purchases, whereas BFI had no significant impact. In another study, Yi, Lee & Jung (2016) preferred MBTI over BFI in personality collaborative filtering recommendation system due to scalability problem associated with BFI when the number of users or items grows.

Myers-Briggs Type Indicator or MBTI is essentially a personality typology using four pairs of traits dichotomy to create 16 personality categories. The four pairs are: Extraversion-Introversion (E-I), Sensing-Intuition (S-N), Thinking-Feeling (T-F), and Judging-Perceiving (J-P). The result is a binary either-or representation from each of the four pairs, which are then combined to make a personality type. For instance, a person more dominant towards Extraversion, Sensing, Thinking and Judging will be of type ESTJ.

#### Significance of Judging-Perceiving dichotomy

Panait & Bucinschi (2018) and Farmer (2018) introduced J/P dichotomy as a representation of the way people function best while dealing with situation, projects and time management. According to Panait & Bucinschi (2018), a person who is a Judging type enjoys being task oriented while planning in advance, while a person who is a Perceiving type prefer to be spontaneous, going with the flow and adapting as event unfolds. J/P dichotomy being only one of the four dichotomies in MBTI, is insufficient to paint out a complete picture of a person’s personality, it is however important by itself to infer certain human behavior that ultimately shape the person status and lifestyle.

Most identified literatures on social media MBTI personality computing had shown a pattern that the Judging-Perceiving (J/P) dichotomy in MBTI is the hardest to predict. Li et al. (2018) suggested that difficulty in predicting J/P dichotomy could be because it involves looking at people’s actions and behaviours, not just words. Result from Lukito et al. (2016) demonstrated that prediction performance on J/P dichotomy does not correlate with number of tweets, and that is it difficult to learn from social media text information. Although Alsadhan & Skillicorn (2017) reported an optimistic accuracy and F1-measure above 80% for J/P dichotomy using an elaborated method, the same method of which source code is not available was attempted without reasonable success. Several other researches have acknowledged that the J/P dichotomy is difficult to be predicted, particularly with textual data (Li et al., 2018; Lukito et al., 2016; Verhoeven, Plank & Daelemans, 2016; Plank & Hovy, 2015; Wang, 2015). Thus, better features are needed for predicting the J/P dichotomy.

Since MBTI is not on a continuous scale, but of four opposing dichotomies, a wrong prediction would mean incorrectly predicting the opposing extreme of a type. This would give a conflicting effect. For instance, a wrong prediction in the J/P dichotomy could mean recommending a career which requires high order of structure like an engineer to a “Perceiving” type person who prefer more creativity and flexibility.

According to a survey conducted by Owens (2015), there is distinctive separations between Judging and Perceiving type participants in their average income and managerial responsibility. Kostelic (2019) studied 244 participants through an online questionnaire and found that J/P dichotomy is a significant variable contributing to one’s decision making and attitude towards solving problem independently or choosing an advisor for help in legal and financial situation. Pelau, Serban & Chinie (2018) acquired 207 valid questionnaires through survey carried out in an urban population, and they found that J/P dichotomy has a significant role on impulsive buying behavior. This result is supported by Yoon & Lim (2018) where the effect of BFI and MBTI on impulsive and compulsive online buying behavior were compared using 296 questionnaires obtained from online shopping mall users in Korea. Yoon & Li (2018) concluded that J/P dichotomy demonstrated a notable impact on both online impulsive and compulsive buying behavior, whereas BFI had no direct impact. In Wei et al. (2017), the authors classified 300 celebrities to their MBTI type and analyzed their respective clothing features from a collection of online images, where they confirmed that J/P dichotomy is significantly correlated to all three clothing features namely color, pattern and silhouette.

Above has demonstrated significance of J/P dichotomy and the impacts it could have on a person not limited to earning power, career responsibilities, inclination to seek help, spending behavior and fashion inclination. These attributes could potentially provide an additional dimension for use cases such as bank loan credibility evaluation, effective personalized advertisement targeting and appropriate career recommendation.

### Feature extraction methods in MBTI prediction

This section describes common feature extraction methods used in MBTI prediction. According to Shah & Patel (2016), feature selection and feature extraction are two methods to solve complexity of machine learning model due to high dimensionality of feature space in text classification. N-gram features are extensively used in text classification. N-gram is a contiguous sequence of n characters or n words within a given n-window where unigram (1-gram) represents individual characters or words, bigram (2-gram) represents two characters or words next to each other, trigram (3-gram) represents three adjacent characters or words. Wang (2015) was able to show different personality behavior through use of bigram in which introvert tend to complain and refuse (“my god”, “holy shit”, “I don’t, I can’t”), while extroverts are more energetic (“so proud”, “can’t wait”, “so excited”).

The bag of words is a simple feature extraction method. The bag of words is essentially the occurrence of word within a defined vocabulary of words, represented thru binary encoding. Simply put, the bag of words checks for the presence of a word within a collection of vocabulary. This bag of words can be constrained to a specific dictionary like in Yamada, Sasano & Takeda (2019) where the author only used words in MeCab’s IPA dictionary. On the other hand, the use of bag of words can be extended with n-gram to include additional words in forms of bigram or trigram as was demonstrated in Wang (2015) and Cui & Qi (2017). In all three researches mentioned, the authors limited the size of the bag of words to n-most frequent words where n is the size defined by the author.

TF-IDF is composed of two parts namely, TF for term frequency and IDF for inverse document frequency. Term frequency measure how frequently a term occurs in a document, whereas inverse document frequency measure how important a term is. TF-IDF is used to evaluate how important a word is to a document. TF-IDF are usually used in combination to other feature extraction methods as a weight to the model. In Alsadhan & Skillicorn (2017), the authors used only term frequency feature alongside with their unique manipulation using digamma function and SVD, outperforming several researches on personality computing. In Gjurković & Šnajder (2018), the best model in the research uses TF-IDF weighted n-grams over logistic regression.

Part-of-speech tagging is a basic form of syntactic analysis where textual data is converted into a list of words, and the words are tagged with their corresponding part-of-speech tag such as whether the word is a noun, adjective, verb, etc. Wang (2015) suggested a correlation between common noun usage and personality where people who uses common noun more often tend to be in extroversion, intuition, thinking, or judging type.

A lexicon is the vocabulary of a language or subject, or simply dictionary words that are assigned to categories to treat them as a set of items with similar context. Individual words can have multiple meaning, thus can belong in multiple categories. A few popular lexical databases are LIWC (Pennebaker et al., 2015), MRC (Wilson, 1988), WordNet (Oram, 2001), Emolex (Mohammad & Turney, 2010). Li et al. (2018) mapped users’ posts into 126 subjectively defined “semantic categories” along with their weights of their categories, and this method was the most successful among other methods in the research for distinguishing all four pairs of dichotomies in MBTI. With the use of LIWC, Raje & Singh (2017) revealed that judging type personality are positively correlated to “work oriented” and “achievement focus” category, and negatively correlated to “leisure oriented”.

Word2Vec is a popular technique to learn word embeddings using shallow neural network that is developed by Mikolov et al. (2013). Word embeddings are vector representation of words where semantic and syntactic relationship between words can measured (Mikolov et al., 2013). Since word embeddings trained on larger datasets perform significantly better, several pretrained word embeddings emerged, for instance older ones like GloVe (Pennington, Socher & Manning, 2014) and more recent ones like ELMo (Peters et al., 2018). In Bharadwaj et al. (2018), the research uses ConceptNet, a pretrained word embeddings and found that it slightly boosted the MBTI prediction performance over original SVM model with TF-IDF+LIWC. Wang (2015) also trained a word2vec model based on an external twitter dataset, they found that the word vectors gave the best individual predictive performance among other features.

Doc2Vec, an extension of Word2Vec where documents are represented as vectors instead of words (Le & Mikolov, 2014). Yamada, Sasano & Takeda (2019) used Distributed Bag of Words (DBOW) model, a type of Doc2Vec but found it to be less effective than the regular Bag of Words model. This is because DBOW is the inferior version of Doc2Vec as compared to the Distributed Memory (DM) version, and usually these two versions are used together for consistency in performance (Le & Mikolov, 2014).

Topic Modeling aims to discover abstract “topics” that occur in a collection of documents. There are several approaches to doing so, the older method is Latent Semantic Analysis (LSA), which is a reduced representation of document based on word term frequency (Landauer, Foltz & Laham, 1998). That is followed by Latent Dirichlet Allocation (LDA) where each document can be described by a distribution of topics and each topic can be described by a distribution of words (Blei, Ng & Jordan, 2003). Gjurković & Šnajder (2018) derived topic distribution from user’s comments using LDA models but found that LDA gave a mediocre performance in MBTI prediction.

### Classification methods used in MBTI prediction

MBTI personality computing is a classification task and thus this section discusses the classification models used in predicting MBTI. Allahyari et al. (2017) and Onan, Korukoğlu & Bulut (2016) identified five of the basic machine learning classifiers, namely: Naïve Bayes, Nearest Neighbour, Support Vector Machine, Logistic Regression, and Random Forest. Lima & Castro (2019) and Raje & Singh (2017) have demonstrated that basic classifier outperformed more advanced classifiers such as ensemble or neural network model in MBTI prediction. However, basic classifiers are suitable for text classification task.

Naïve Bayes classifier models the distribution of documents in each class using a probabilities model with assumption that the distribution of different terms is independent from each other. Naive Bayes classifier is computationally fast and work with limited memory. Multinomial Naïve Bayes model is commonly used for text classification task. However, Rennie et al. (2003) discourages the use of Multinomial Naïve Bayes model stating that it does not model text well. Instead they introduced Complement Naïve Bayes as a emulate a power law distribution that matches real term frequency distribution more closely. Celli & Lepri (2018) uses AutoWeka, a meta classifier that automatically find the best algorithm and setting for the task. AutoWeka had particularly chosen Naïve Bayes as the classifier of choice in prediction the Judging-Perceiving dichotomy.

Nearest Neighbour classifier is a proximity-based classifier which use distance-base measures to perform classification. k is referred as the number of neighbours to be considered., the most common class among k-Neighbours will be reported as the class label. Nearest Neighbour classifier is not particularly a popular classifier for text classification task. Only one research, Li et al. (2018) on MBTI prediction were found to be using this classifier without any comparison to other classifiers. The main goal of that research though is to compare and find the best distance computation methods for KNN in MBTI personality computing.

Support vector machine finds a “good” linear separator between various classes based on linear combinations of the documents features. Kernel techniques are used in SVM to transform linearly inseparable problems into higher dimensional space. Commonly used kernels are Radial Basis Function, Polynomial kernel and Gaussian function. It is important to note that while SVM run time is independent of the dimensionality of the input space, it scales with the number of data points with a time complexity of O(n^3^) (Abdiansah & Wardoyo, 2015). SVM classifier is a popular classifier for text classification tasks. Bharadwaj et al. (2018) compared SVM to Naïve Bayes and Neural Net classifiers in MBTI prediction and saw that SVM outperformed the other classifiers across three feature vector sets.

Logistic Regression takes the probability of some event’s happening and model it as a linear function of a set of predictor variables (Onan, Korukoğlu & Bulut, 2016). It only works with binary classes. Raje & Singh (2017) used Logistic Regression for the MBTI prediction task and found that the performance of the classifier slightly outperforms Artificial Neural Network (ANN) classifier. Gjurković & Šnajder (2018) compared three classifiers and saw that Logistic Regression outperforms SVM on MBTI prediction.

Random Forest is an ensemble of many randomized decision trees, where each decision trees gives a prediction to the task and the results are averaged or the major vote is selected to be the final prediction. Lima & Castro (2019) found that Random Forest consistently outperforms SVM and Naïve Bayes classifiers across multiple feature sets by a large degree in MBTI prediction.

Similar to Random Forest, Gradient Boosting Decision Tree (GBDT) is an ensemble model of decision trees but is trained in sequence for which each iteration GBDT learns the decision tree by fitting the negative gradients (Ke et al., 2017). A popular variant of GBDT is XGBoost, which has a track record of outperforming other ML models as observed on challenges hosted on a machine learning competition platform Kaggle (Chen & Guestrin, 2016). A more recent development is LightGBM, the authors Ke et al. (2017) demonstrated two features in LightGBM namely gradient-based one-side sampling and exclusive feature bundling that enabled LightGBM to significantly outperform XGBoost especially when training a model with sparse features. Although popular, none of the GBDT methods were found to be used in identified related work.

Table 1 shows a compilation of all identified related work along with their best classifier, features and performance metrics. Among all seventeen researches mentioned in Table 1, only nine were using overlapping datasets from three publicly available dataset. Two of these datasets were from Twitter and one was from Personality Café Forum. With the exception of Gjurković & Šnajder (2018), the rest of the researches uses private dataset and their method was not tested on publicly available dataset for comparison. This makes it difficult to tell how well their method fare among the other researches’ method.

**Table 1:** Research on MBTI prediction from social media textual data.

Research	Dataset	Best classification methods	Feature extraction methods	Metric	E/I	S/N	T/F	J/P
Brinks & White (2012)	Twitter	Naïve Bayes	TF, IDF	Accuracy	63.90%	74.60%	60.80%	**58.50%**
Plank & Hovy (2015)	Twitter^a^	Logistic Regression	n-grams, Metadata	Accuracy	72.50%	77.40%	61.20%	**55.40%**
Wang (2015)	Twitter	Logistic Regression	POS, n-gram, word vector, BOW	AUC	69.10%	65.30%	68.00%	**61.90%**
Lukito et al. (2016)	Twitter	Naïve Bayes	n-gram	Accuracy	80.00%	60.00%	60.00%	**60.00%**
Verhoeven, Plank & Daelemans (2016)	Twitter^b^	LinearSVC	n-gram	F-measure	67.87%	73.01%	58.45%	**56.06%**
Cui & Qi (2017)	Kaggle^c^	LSTM	n-gram, BOW, POS, capital letter count	Accuracy	89.51%	89.85%	69.10%	**67.65%**
Raje & Singh (2017)	Twitter	Logistic Regression	LIWC	Accuracy	53.57%	51.99%	56.25%	**53.57%**
Alsadhan & Skillicorn (2017)	Twitter^a^	SVD component distance threshold	TF	Accuracy	90.00%	92.00%	80.00%	**83.00%**
				F-measure	90.0%	92.00%	80.00%	**84.00%**
	Twitter^b^	SVD component distance threshold	TF	Accuracy	82.67%	81.84%	76.83%	**80.50%**
				F-measure	82.33%	86.67%	78.17%	**81.17%**
Bharadwaj et al. (2018)	Kaggle^c^	SVM	BOW, TF-IDF, LIWC, EmoSenticNet, ConceptNet	Accuracy	84.90%	88.40%	87.00%	**78.80%**
Li et al. (2018)	Kaggle^c^	K Nearest Neighbor	Word category, Nuance, TF, IDF,TF-IDF	Accuracy	90.00%	90.00%	91.25%	**76.25%**
Celli & Lepri (2018)	Twitter	SVM	n-gram, LIWC, Metadata	Accuracy	61.30%	68.50%	68.60%	**60.20%**
Keh & Cheng (2019)	Personality Cafe Forum	BERT	Pretrained BERT model	Accuracy	75.83%	74.41%	75.75%	**71.90%**
Gjurković & Šnajder (2018)	Reddit^d^	Multilayer Perceptron	n-gram, TF-IDF, LIWC, MRC Metadata, temporal features	F-measure	82.80%	79.20%	64.40%	**74.00%**
Yamada, Sasano & Takeda (2019)	Twitter	SVM	BOW, DBOW, Metadata	AUC	73.18%	69.89%	70.96%	**62.10%**
Lima & Castro (2019)	Twitter^a^	Random Forest	LIWC, oNLP	Accuracy	82.05%	88.38%	80.57%	**78.26%**
				F-measure	78.75%	82.40%	79.25%	**77.58%**
				AUC	86.94%	87.16%	87.94%	**88.02%**
Amirhosseini & Kazemian (2020)	Kaggle^c^	XGBoost	TF-IDF	Accuracy	78.17%	86.06%	71.78%	**65.70%**
Mehta et al. (2020)	Kaggle^c^	BERT + MLP	Pretrained BERT model,LIWC, SenticNet, NRC Emotion Lexicon, VAD Lexicon, Readability	Accuracy	78.80%	86.30%	76.10%	**67.20%**

**Notes.**

a Dataset available on https://bitbucket.org/bplank/wassa2015/src/master/.

b Dataset available on https://www.uantwerpen.be/en/research-groups/clips/research/datasets/.

c Dataset available on https://www.kaggle.com/datasnaek/mbti-type.

d Dataset available by request on http://takelab.fer.hr/data/mbti.

As far as classification methods used, basic machine learning models were predominantly used in researches found. Only four out of seventeen researches utilized deep learning algorithm in their method. A few feature extraction methods were heavily used such as term frequency, n-gram and word categories like LIWC. Only two researches utilized word embeddings, and one used BERT sequence classification alone on raw sentences without any feature extraction.

Accuracy is the most used metric for these researches, however this is not the best metric to use for imbalance data. For example, in E/I dichotomy, if the distribution of class E is 80% and class I is 20%, predicting all samples as class E will yield 80% accuracy anyhow although class I is predicted wrongly 100% of the time. For dataset that are not openly available, there is no way to gauge the actual performance of the method in the research as the distribution of the classes is sometimes not specified.

## Methodology

**Figure 1:**
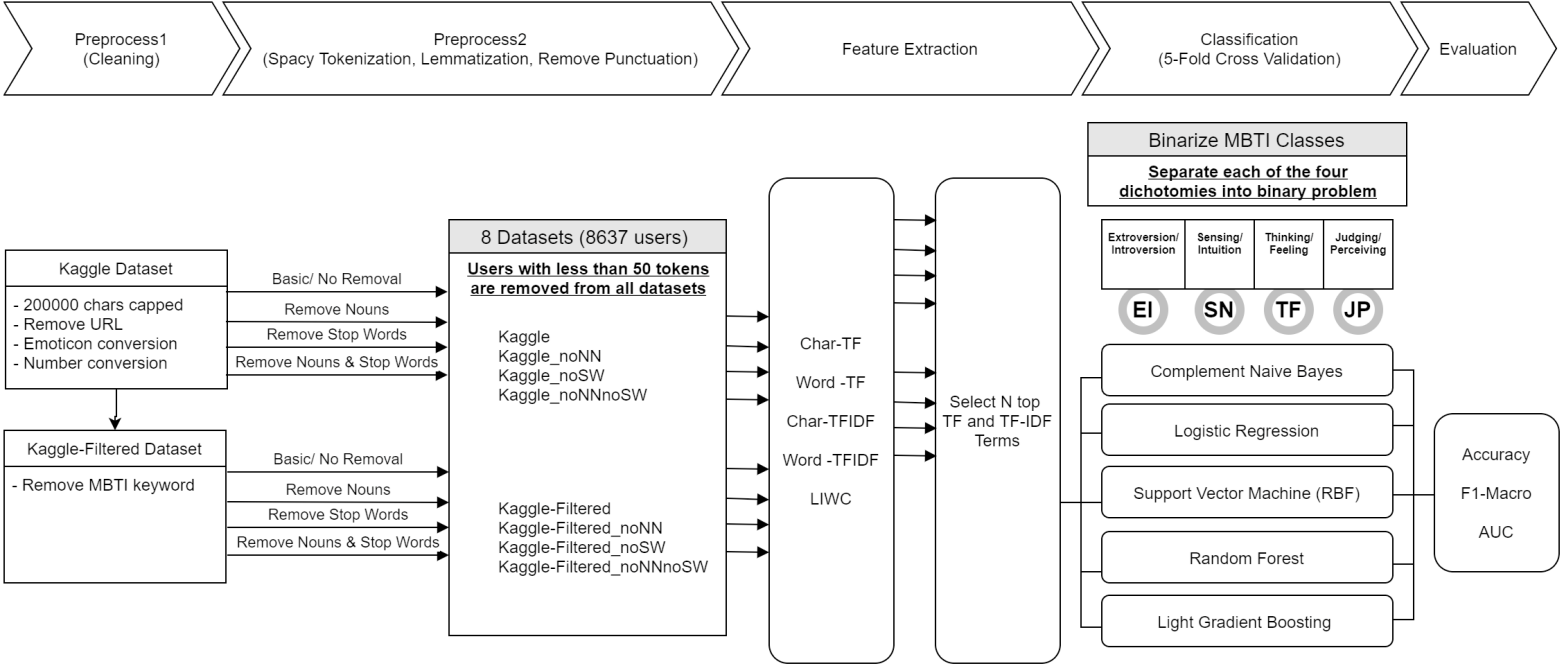
Research framework.

This research followed a customized framework for approaching MBTI’s Judging-Perceiving dichotomy classification on online social forum dataset as shown in Fig. 1. The preprocessing is in two stages, stage one deal with cleaning of the data such as removing duplication, standardizing the inputs, etc. Stage two performs the necessary tokenization along with lemmatization and punctuation removal. Additional preprocessing was performed such as stop word or noun removal to create a diverse dataset for comparison of the effect of these data processing methods. After preprocessing, features were extracted from datasets using several methods, which are discussed in detail in ‘Feature extractions and dimensionality reduction’. Subsequently under classification stage, the features and labels were fed into a machine-learning model for training and prediction. Finally, in evaluation, the metric results were evaluated.

### Tools and resources

This research was conducted on Windows 10 using Python, a high-level scripting language in conjunction with Jupyter Notebook. Various open source Python libraries were used for this research, and they will be detailed in the following sections. Linguistic Inquiry and Word Count, LIWC 2015 by Pennebaker et al. (2015) is the only paid service subscribed in this research to replicate some of the work that had been done with the same dataset that this research was conducted on. For the purpose of reproducibility, all source codes were documented and made available on Github: https://github.com/EnJunChoong/MBTI_JP_Prediction.

### Dataset

The dataset used in this research is referred as Kaggle dataset. The Kaggle dataset was crawled from Personality Cafe forum in 2017, it consists of the last 50 posts made by 8675 people, whom MBTI type were known from their discussion on this online social forum platform (Li et al., 2018). Using a simple white space tokenization, each person in the dataset has an average of 1226 words with standard deviation of 311 words. A further dive into the numbers found each post contains average of 26 words with a standard deviation of 13 words. Personality Cafe forum is a forum for conversation and discussion on personality. The Kaggle dataset is available on http://www.kaggle.com, which is a website dedicated to data science enthusiasts. The dataset only contained two columns, one is the user MBTI type, and another is their respective posts on Personality Cafe forum. Five researches on the same dataset were identified, namely Cui & Qi (2017), Bharadwaj et al. (2018), Li et al. (2018), Mehta et al. (2020) and Amirhosseini & Kazemian (2020). The justifications on the reasons of choosing this dataset are as follows:

(1) Publicly available dataset of substantial size.
(2) Dataset which is not based on microblogs (microblog data needs to be handled differently)
(3) Cited at least twice to establish reference for comparison.

**Figure 2:**
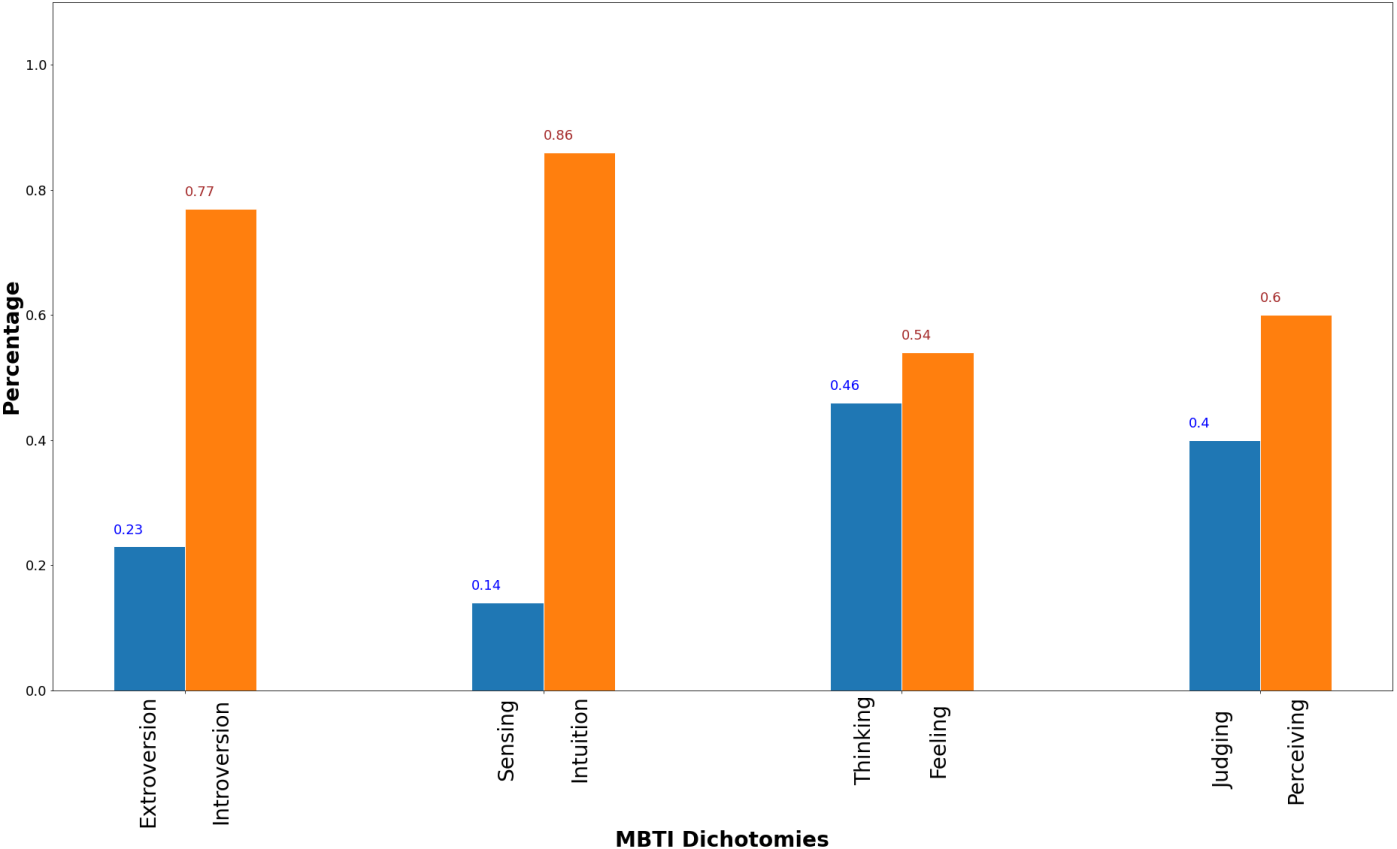
MBTI dichotomies dataset distribution.

Figure 2 shows the dataset MBTI dichotomies distribution. The E/I is the most imbalance pair among the four followed by and S/I pair, then J/P pair and lastly T/F pair. At a 4:6 ratio for Judging to Perceiving, the J/P pair distribution is much more balanced than the distribution of E/I and S/I pairs. However, a more balanced dataset does not signify better prediction performance as shown in Table 1 where prediction performance on J/P pair is worse than E/I and T/F pairs for three related works that uses the same dataset denoted as Kaggle^3^.

### Data preprocessing

**Figure 3:**
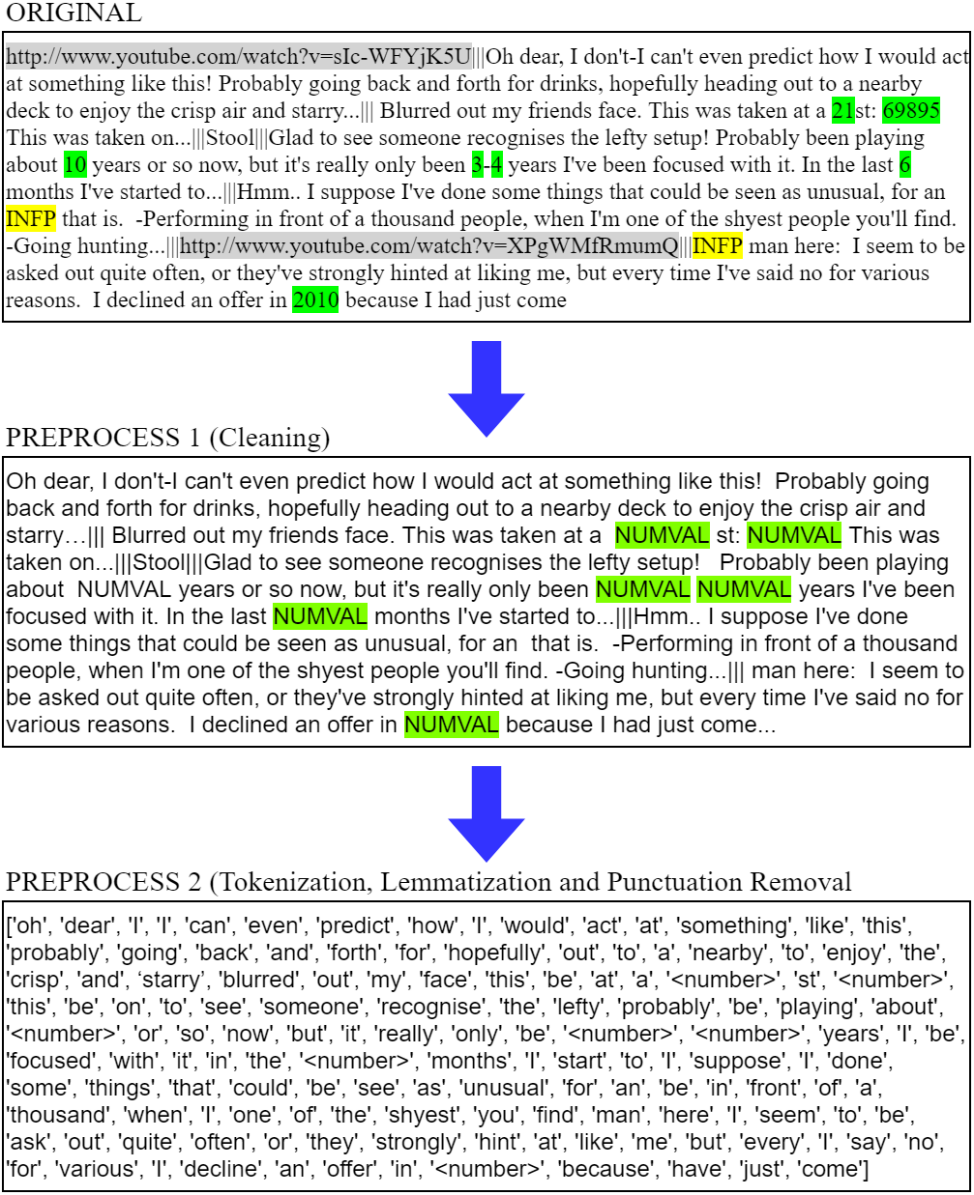
Preprocessing example.

This research separated preprocessing into two parts. Part one dealt with data cleaning and wrangling, which is the removal of messy erroneous data. Then part two was the tokenization of the textual data, which is the transformation into structured machine comprehensible data. Figure 3 is an example of the transformation of the original data to the final tokens. It can be observed that URLs shaded in gray and MBTI keywords shaded in yellow were removed in first phase of preprocessing. And numbers shaded in green are replaced with an escape word. “NUMVAL”. Subsequently the data is tokenized, lemmatized and removed for punctuation. In this example, additional stop word and nouns were removed as well.

#### Data cleaning

For data cleaning, both datasets were capped at 200,000 characters per user. Regular expression was utilized to remove URLs, to convert emoticons into 4 categories (*smileface*, *lolface*, *sadface* and *neutralface*), and to convert any numeric digits into a category called *numval*. The Kaggle dataset is highly bias towards personality discussion, and very often the posts contain MBTI keywords. Cui & Qi (2017), Bharadwaj et al. (2018) and Li et al. (2018) did not mention removal of the MBTI keywords in their research on Kaggle dataset. On a similar dataset, Keh & Cheng (2019) scraped 68,000 posts from Personality Cafe Forum, and they explicitly removed the MBTI keyword. There is no absolute way to tell the effect of the MBTI keyword bias on the dataset. Thus, a new Kaggle-Filtered dataset was introduced where all 16 MBTI keywords were removed. The removal of MBTI keyword was done using regular expression.

#### Tokenization, lemmatization and punctuation removal

Tokenization was done with spaCy library using its’ pretrained medium size English model. The two datasets: Kaggle, Kaggle-Filtered were sent to spaCy for tokenization and lemmatization. Punctuation was removed by default for all dataset during this process. Common perception on stop words are that they are of little use and not informative (Wang, 2015). However, Plank & Hovy (2015) stated that stop word removal harms performance, and Alsadhan & Skillicorn (2017) argued that stop words are predictive of authorship and so of individual differences, the latter suggested that noun is to be removed instead. To investigate this problem, four versions for each of the dataset were introduced as follow:

• No removal of noun or stop words
• Removal of noun
• Removal of stop words
• Removal of noun and stop

In order to have enough representation of word tokens per user, users with less than 50-word tokens were identified and removed from the dataset group. Only Kaggle dataset were reduced from 8675 users to 8637 users. At this point, from the initial 2 datasets, additional datasets were generated to a total of 8 datasets at the end.

### Feature extractions and dimensionality reduction

For each of the 8 datasets, the authors extracted a set of linguistic features from the users’ post or comments on the online social forum. A few popular feature extraction methods for social media MBTI prediction were identified, among them are TF-IDF, part of speech, word embeddings and word categories. Char-level TF-IDF, word-level TF-IDF, and LIWC were decided to be used as the research main feature extraction methods. The implementation of these features will be discussed in the following subsections.

An extra transformation step was done for the features before feeding them to the classification task. In Python, the authors selected scikit-learn’s preprocessing module called QuantileTransformer to transform all features into following a uniform distribution that range between 0 and 1. This method collapses any outlier to the range boundaries and is less sensitive to outlier than the common standard scaling or min-max scaling method.

#### Character-level TF and TF-IDF

The main purpose of TF and TF-IDF is to simply give a heavier weight to terms that have the highest likelihood to distinguish a document from the others. While word-level is a more common feature extraction method, Gjurković & Šnajder (2018) demonstrated that character-level TF has more relevant features than word TF, and that TF are generally better than TF-IDF in MBTI personality computing.

\CountVectorizer and TfidfVectorizer module of Sci-kit learn were used to perform the computation for character level TF and TF-IDF. The ngram parameter used were 2–3 ngram. However, it still takes whitespace as a gram if the adjacent character is at the edge of the word. For illustration:

**Table:** 

Sentence:	‘I am a bear’
Character ngram:	‘I’, ‘a’, ‘am’, ‘a’, ‘b’, ‘be’,’bea’, ‘ea’, ‘ear’, ‘ar’, ‘r’

The authors capped the number of terms to 1500 and eliminate terms that have appearances less than 50 documents or more than 95% of the entire corpus.

#### Word-Level TF-IDF

Like the character-level TF and TF-IDF, similar logic was applied using the same TF and TF-IDF feature extraction method. This time however, word level instead of character level was used. The ngram parameter used will be from 1 to 3 since a word by itself could have much more importance as compared to a single alphabet in a big corpus. Here, the illustration is much simpler.

**Table:** 

Sentence:	‘I am a bear’
word ngram:	‘I ‘, ‘am’, ‘a’, ’bear’, ‘I am’, ‘am a’, ‘a bear’, ‘I am a’, ‘am a bear’

The authors capped the number of terms to 1500 and eliminate terms that have appearances less than 50 documents or more than 95% of the entire corpus.

### Classification and model validation

Logistic regression (LR), Complement Naïve Bayer (CNB), Support Vector Machine (SVM) and Random Forest (RF) were selected for their popularity and effectiveness in text classification problems (Gjurković & Šnajder, 2018; Rennie et al., 2003; Bharadwaj et al., 2018; Lima & Castro, 2019). LightGBM (LGB), a gradient boosting framework that uses tree-based learning algorithms was also added to the list of classifiers to provide an additional comparison. LightGBM was chosen instead of XGBoost due to the model effectiveness in dealing with sparse features as elaborated in ‘Data preprocessing’.

Like any classification task, the target variables and features need to be clearly defined. From the feature extraction step, following feature sets are obtained:

• Character-level TF (1500 attributes)
• Character-level TFIDF (1500 attributes)
• Word-level TF (1500 attributes)
• Word-level TFIDF (1500 attributes)
• LIWC (78 attributes)
• Combo (Combination of all features which comprise of Character-level TF, Character- level TFIDF, Word-level TF, Word-level TFIDF and LIWC) (6078)

**Figure 4:**
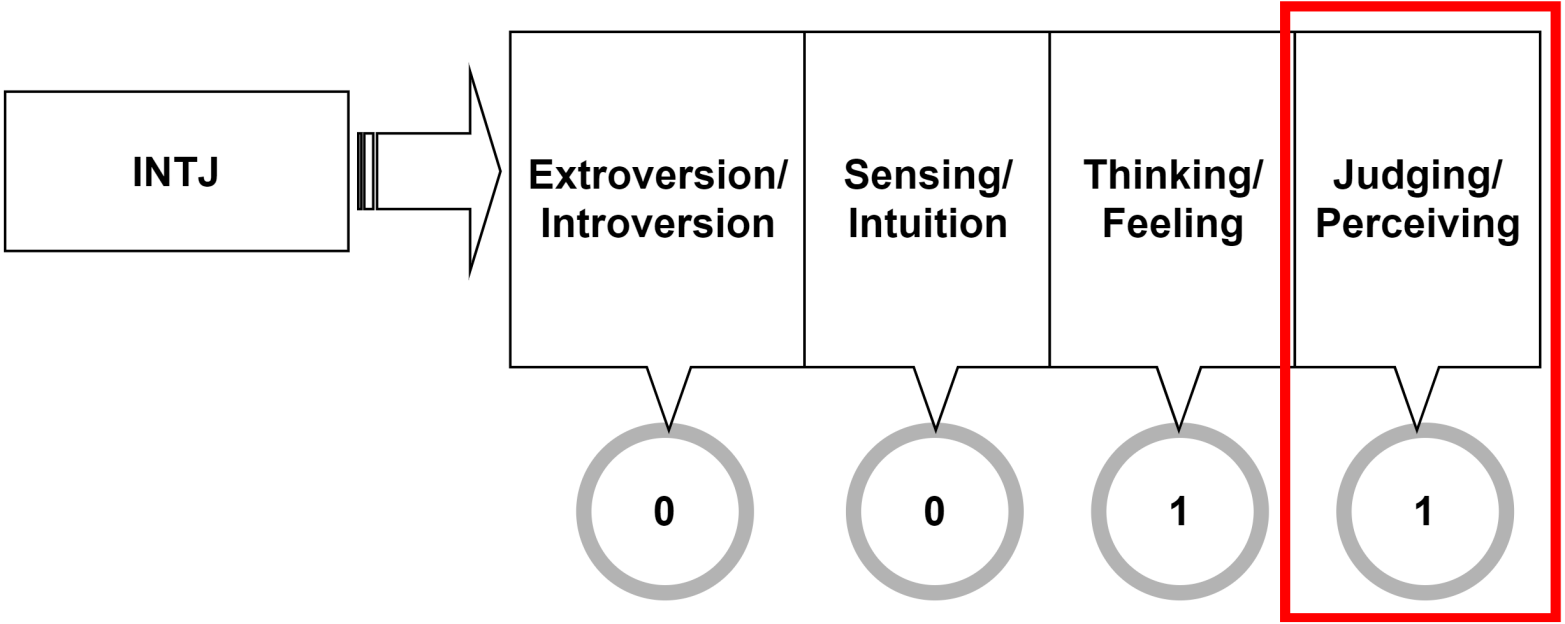
Target variable binarization.

Since the research focus is on predicting MBTI’s judging and perceiving dichotomy, the problem must be binarized. For each user, their MBTI type is split into four dimensions accordingly to the four MBTI dichotomies. Figure 4 illustrates that the last column, bounded in the box is corresponding to Judging-Perceiving dichotomy, and this is the only column this research is interested in. In this research, judging/perceiving class is assigned the value of 1 in the label.

Once target variable and features sets were clearly defined, they are sent through a set of classifiers within a stratified 5-fold cross validation. While most researches use a simple 5-fold cross validation, this research use stratified 5-fold cross validation to maintain the distribution of classes among training and testing dataset.

## Results & Discussion

### Results

This section states the results obtained in this study. The prediction performance of the five proposed classifiers were evaluated based on the accuracy and F1-Macro score. Since accuracy metric is more sensitive to the distribution of the target variable, F1-Macro score is evaluated on top of accuracy since it is more important to capture the sensitivity and specificity performance of a classifier on an imbalance dataset.

The accuracy and F1-Macro score for the classifiers on Kaggle and Kaggle-Filtered dataset is tabulated in Table 2. According to Table 2, LightGBM classifier on average perform significantly better than the other classifiers on Kaggle dataset. However, in Kaggle-Filtered, LightGBM only slightly outperformed other classifiers.

**Table 2:** Accuracy and F1-Macro score for Kaggle and Kaggle-Filtered dataset.

Dataset	Models	Accuracy (%)					F1-Macro (%)				
		CNB	LGB	LR	RF	SVM	CNB	LGB	LR	RF	SVM
**kaggle**	**combo**	75.58	**81.68**	69.19	78.14	80.13	74.57	**80.77**	68.1	75.04	79.21
	**char_tf**	67.23	**81.66**	73.16	76.30	75.20	66.52	**80.76**	72.31	72.16	74.44
	**char_tfidf**	67.13	**81.22**	73.60	76.14	75.58	66.44	**80.29**	72.76	71.89	74.86
	**word_tf**	68.94	**80.17**	69.70	71.18	73.35	68.16	**79.2**	68.83	63.78	72.33
	**word_tfidf**	68.80	**79.90**	69.85	70.75	73.35	68.04	**78.94**	68.99	63.17	72.37
	**LIWC**	56.65	57.4	58.16	**60.69**	57.84	55.98	56.23	**57.45**	46.74	57.18
		**CNB**	**LGB**	**LR**	**RF**	**SVM**	**CNB**	**LGB**	**LR**	**RF**	**SVM**
**kaggle-Filtered**	**combo**	61.22	**66.26**	59.28	62.01	64.04	60.27	**63.83**	58.04	45.84	62.84
	**char_tf**	60.02	**63.77**	60.38	61.16	62.57	59.19	61.38	59.44	42.66	**61.57**
	**char_tfidf**	59.82	**64.50**	60.50	61.36	62.31	59.08	**62.01**	59.55	43.82	61.47
	**word_tf**	61.13	**65.80**	60.30	61.22	61.76	60.33	**63.99**	59.32	42.7	60.74
	**word_tfidf**	60.95	**64.85**	60.02	61.20	61.63	60.13	**62.75**	59.03	44.09	60.83
	**LIWC**	56.58	57.08	58.19	**60.65**	58.02	55.88	55.99	**57.51**	46.46	57.34

**Notes.**

- bold values are highest value across classifiers.

- combo is the combination of char_tf, char_tfidf, word_tf, word_tfidf and LIWC.

**Figure 5:**
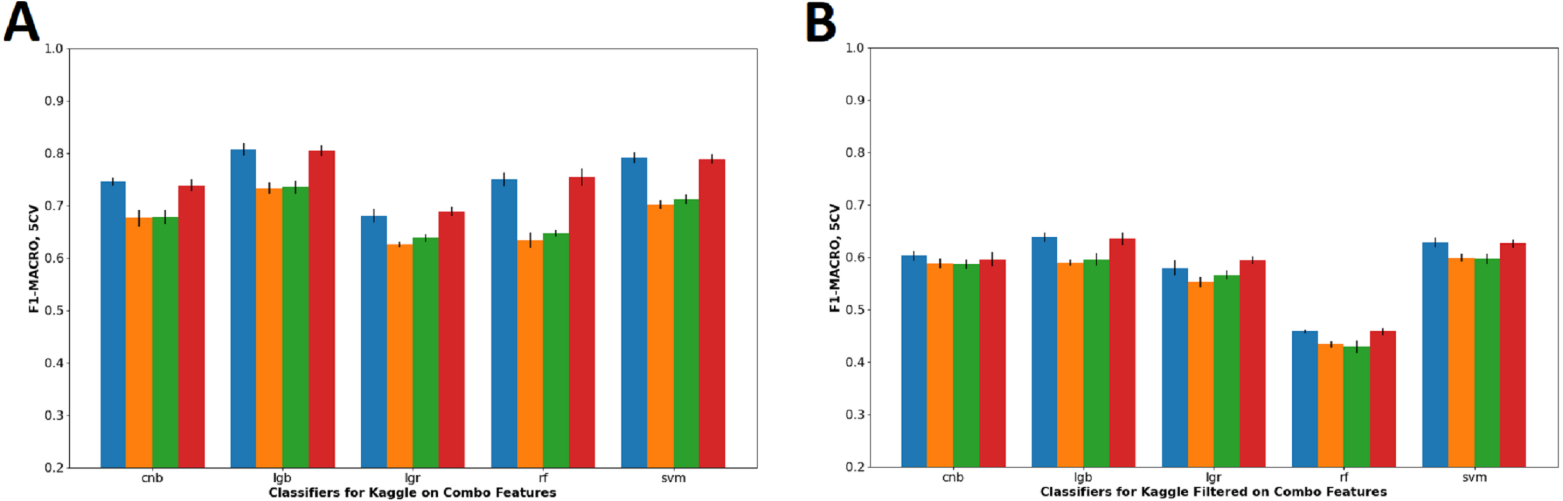
Visualization of combo features F1-Macro score on all dataset configuration.

Table 3 shows the accuracy and F1-Macro score of classifiers on combo feature set. Combo feature set were chosen for comparison because combo feature set is inclusive of the other feature sets and thus can generalize the effect of the noun and stop words removal. Based on the result in Table 3 and corresponding visualization in Fig. 5, both depicts that the removal of noun words drastically reduces prediction performance of J/P dichotomy in Kaggle dataset, whereas slightly to no effect on Kaggle-Filtered dataset. The removal of stop words has negligible effect on the prediction performance in all datasets.

**Table 3:** Accuracy and F1-Macro score for all datasets on combo feature set.

Datasets	Accuracy on combo feature set (%)					F1-Macro on combo feature set (%)				
	cnb	lgb	lgr	rf	svm	cnb	lgb	lgr	rf	svm
Kaggle	75.58	**81.68**	69.19	78.14	80.13	74.57	**80.77**	68.1	75.04	79.21
Kaggle_noNN	68.6	**74.57**	63.97	70.33	71.43	67.62	**73.34**	62.65	63.43	70.22
Kaggle_noNNnoSW	68.82	**74.86**	64.98	70.96	72.37	67.8	**73.53**	63.82	64.69	71.25
Kaggle_noSW	74.96	**81.41**	70.01	78.42	79.82	73.89	**80.51**	68.93	75.47	78.94
Kaggle-Filtered	61.22	**66.26**	59.28	62.01	64.04	60.27	**63.83**	58.04	45.84	62.84
Kaggle-Filtered_noNN	59.66	**61.47**	56.66	61.09	60.95	58.87	59.00	55.26	43.35	**59.89**
Kaggle-Filtered_noNNnoSW	59.59	**61.83**	57.99	60.81	60.61	58.69	59.59	56.67	42.98	**59.67**
Kaggle-Filtered_noSW	60.68	**65.89**	60.65	61.90	63.78	59.60	**63.56**	59.38	45.84	62.66

**Notes.**

- bold values are highest value across classifiers and datasets.

- noNN = Noun removed, noSW = Stop Words removed, noNNnoSW = Noun and Stopwords removed.

- combo is the combination of char_tf, char_tfidf, word_tf, word_tfidf and LIWC.

The combination of LightGBM and Combo Feature on Kaggle dataset produces the highest F1-Macro score of 80.77%. Table 4 tabulates the confusion matrix, recall, precision and f1-measure from the results of the 5-folds cross-validations. Visibly, the recall, precision and f1-measure for predicting Perceiving class is above 8–10% higher than predicting Judging class. This suggest that people with Perceiving traits have more prominent linguistic marker than people with Judging trait when it comes to communicating on social media.

**Table 4:** 5-folds cross-validation results of Kaggle dataset under best configuration: LightGBM and Combo features.

Confusion matrix					Performance metric score (%)				
5-folds cross validation			Predicted class		Accuracy	Recall	Precision	F1-Measure	AUROC
			Judging	Perceiving					
**Fold-1**	**Actual Class**	**Judging**	522	162	82.41	76.32	78.61	77.45	89.03
		**Perceiving**	142	902		86.4	84.77	85.58	
**Fold-2**		**Judging**	531	153	82.23	77.63	77.52	77.57	90.32
		**Perceiving**	154	890		85.25	85.33	85.29	
**Fold-3**		**Judging**	503	180	79.97	73.65	75.19	74.41	86.99
		**Perceiving**	166	878		84.1	82.99	83.54	
**Fold-4**		**Judging**	513	170	81.24	75.11	76.91	76.00	88.60
		**Perceiving**	154	890		85.25	83.96	84.60	
**Fold-5**		**Judging**	518	165	82.57	75.84	79.20	77.48	89.37
		**Perceiving**	136	908		86.97	84.62	85.78	
**Average/ Macro-Average**					81.68	80.65	80.91	80.77	88.86
**Standard Deviation**					1.09	1.13	1.17	1.14	1.22

**Table 5:** Results comparison with past researches.

Methods	Dataset	Performance metric score ± Standard deviation (%)			Best configuration
		Accuracy	F1-Macro	AUROC	
Cui & Qi (2017)	Kaggle	62.65%	NA	NA	NA
Bharadwaj et al. (2018)	Kaggle	78.80%	NA	NA	NA
Li et al. (2018)	Kaggle	76.25%	NA	NA	NA
Amirhosseini & Kazemian (2020)	Kaggle	65.70%	NA	NA	NA
Mehta et al. (2020)	Kaggle	67.20%	NA	NA	NA
**Light GBM (This research)**	**Kaggle**	**81.68**±**1.09%**	**80.77± 1.14%**	**88.86± 1.22%**	**Combo**

The results for all experimental configurations are tabulated in Appendix A: Tables A1 and A2 detail the 5-fold cross validation results average; whereas Tables A3 and A4 detail the results standard deviation for Kaggle and Kaggle-Filtered dataset respectively.

### Benchmarking with previous researches

Compiling all results from this research, and the result from relevant research using the same dataset, Table 5 shows a comparison of this research with those of the past. Without addressing the data leakage in Kaggle dataset, Light GBM model with character-level TF managed to outperform J/P prediction performance of past researches on the same dataset. However, that cannot be reproduced with Kaggle-Filtered dataset with MBTI keyword removed. Four researches, Cui & Qi (2017), Bharadwaj et al. (2018), Li et al. (2018) and Mehta et al. (2020) did not mention explicitly about removal of MBTI keyword in their preprocessing step. Only Amirhosseini & Kazemian (2020) mentioned removal of MBTI keywords in their preprocessing steps.

Referring to Table 1 in the related works section, best performing algorithm from Cui & Qi (2017), Bharadwaj et al. (2018), Li et al. (2018), Mehta et al. (2020) and Amirhosseini & Kazemian (2020) are long short-term memory (LSTM), support vector machine (SVM), k nearest neighbor (KNN), BERT + MLP and XGBoost. LightGBM used in this research achieved 81.68% accuracy with a standard deviation of 1.09%, and had outperformed the accuracy of all previous work on this dataset as indicated in Table 5.

It is not surprising the LightGBM came up above SVM and KNN since most competition on Kaggle were won using gradient boosting algorithm such as LightGBM and XGboost. Unexpectedly, LSTM, a deep learning algorithm was the lowest among all. There is no concrete explanation to this, but it is worth nothing that Cui & Qi (2017) had remediated the data so that no one class out of the 16 MBTI type will be twice as large as the other. Thus, the final distribution of the classes is different, and the distribution were not mentioned in the research. Additionally, Cui & Qi (2017) were training the LSTM for a multi class problem to output 16 MBTI types, thus the model is not learning predominantly for judging/perceiving dichotomy. All being said, comparison of the results using accuracy as the metric on Cui & Qi (2017) is not valid.

## Conclusion & Future Work

This research had demonstrated that there is negligible difference in social media MBTI Judging-Perceiving prediction performance between character-level TF, TF-IDF and word-level TF, TF-IDF. LIWC is consistently behind by a small margin in prediction. Word-level features are recommended over character-level feature for the better interpretability and marginally higher predictive power.

Five classifiers were compared in the task of MBTI Judging-Perceiving prediction. While both SVM and LightGBM are clearly superior as compared to other three classifiers, the prediction performance of LightGBM and SVM are rather similar. This research recommended LightGBM in the end for a better robustness in achieving convergence as SVM failed to do so in one of the datasets.

On the final objective, this research evaluated the J/P dichotomy prediction performance of all eight datasets. The highest F1-Macro score across the two groups for Kaggle and Kaggle-Filtered datasets are 81% and 65%, respectively. The importance of proper preprocessing is illustrated by showing the contrast in prediction performance between Kaggle, a dataset with data leakage and Kaggle-Filtered, a dataset without data leakage. . This suggest that J/P dichotomy prediction might rely heavily on the linguistic semantic rather than statistical approach on the terms like TF and TF-IDF. A semantic-based approach would be necessary for tackling the prediction of MBTI J/P dichotomy.

Past researchers raised a valid point that the posts drawn from Personality Cafe could lead to many inherent data biases since users and posts are only sampled from one forum with discussion revolving a focused topic. This cannot be generalized to users on another forum as the topic diversity is too narrow. Although some researchers have provided a social media MBTI corpus from reddit with a great topic diversity, it does not shy away from the fact that the classes of the users represented on the corpus are largely imbalance and is nowhere near the realistic distribution. Yet, another problem with the current available large corpuses is that the MBTI label from these corpuses are from self-administered MBTI assessment. There is no information on which version of MBTI assessment was used thus producing inconsistent data. Coming up with a validated corpus with a better representation of the MBTI distribution remains to be the number 1 task to be pursued.

## Appendix A

See Table A1–A4

**Table A1:** Five-fold cross validation result average for Kaggle dataset without MBTI keyword removed.

Features	Dataset	Kaggle					Kaggle_noNN					Kaggle_noNNnoSW					Kaggle_noSW				
	Models	cnb	lgb	lgr	rf	svm	cnb	lgb	lgr	rf	svm	cnb	lgb	lgr	rf	svm	cnb	lgb	lgr	rf	svm
**combo**	**Acc**	75.58	81.68	69.19	78.14	80.13	68.60	74.57	63.97	70.33	71.43	68.82	74.86	64.98	70.96	72.37	74.96	81.41	70.01	78.42	79.82
	**F1-macro**	74.57	80.77	68.10	75.04	79.21	67.62	73.34	62.65	63.43	70.22	67.80	73.53	63.82	64.69	71.25	73.89	80.51	68.93	75.47	78.94
	**AUROC**	81.51	88.86	74.68	86.83	86.82	74.08	81.25	67.75	77.82	77.79	74.04	81.53	69.34	78.80	78.26	80.96	88.80	76.03	87.08	86.83
	**Precision-macro**	74.48	80.91	68.00	80.46	79.24	67.49	73.45	62.59	73.81	70.18	67.68	73.76	63.74	74.05	71.18	73.83	80.59	68.81	80.49	78.89
	**Recall-macro**	74.68	80.65	68.31	74.02	79.19	67.93	73.29	62.78	64.05	70.30	68.08	73.37	64.05	65.00	71.37	73.96	80.44	69.13	74.44	79.00
**char_tf**	**Acc**	67.23	81.66	73.16	76.30	75.20	66.01	74.25	66.53	69.33	69.86	65.57	73.81	66.23	68.77	69.71	66.61	81.16	73.09	75.28	75.01
	**F1-macro**	66.52	80.76	72.31	72.16	74.44	65.22	73.00	65.67	61.63	68.94	64.80	72.59	65.37	60.89	68.83	65.87	80.29	72.26	70.78	74.26
	**AUROC**	72.91	88.50	80.60	85.19	82.57	71.30	80.72	72.47	76.40	75.92	70.71	80.69	71.91	75.44	75.60	72.22	88.44	80.21	85.04	82.34
	**Precision-macro**	66.49	80.88	72.14	79.83	74.23	65.18	73.12	65.60	73.08	68.79	64.78	72.66	65.31	72.08	68.70	65.84	80.32	72.08	78.83	74.07
	**Recall-macro**	67.12	80.68	72.66	71.37	74.85	65.73	72.95	66.12	62.71	69.28	65.34	72.57	65.82	62.11	69.22	66.44	80.27	72.64	70.15	74.71
**char_tfidf**	**Acc**	67.13	81.22	73.60	76.14	75.58	65.97	74.35	66.61	68.98	69.70	65.75	73.81	66.30	69.12	69.40	66.57	80.95	73.03	76.29	75.19
	**F1-macro**	66.44	80.29	72.76	71.89	74.86	65.20	73.08	65.75	60.92	68.84	65.02	72.48	65.41	61.26	68.54	65.90	80.03	72.20	72.20	74.48
	**AUROC**	73.09	88.43	80.66	85.42	82.84	71.23	80.42	72.64	76.71	75.92	70.74	80.36	71.93	76.70	75.66	72.40	88.26	80.26	85.20	82.63
	**Precision-macro**	66.43	80.42	72.58	79.84	74.65	65.16	73.21	65.68	72.92	68.70	65.02	72.65	65.34	72.79	68.41	65.91	80.12	72.03	79.70	74.30
	**Recall-macro**	67.07	80.19	73.11	71.13	75.33	65.73	73.00	66.19	62.21	69.25	65.60	72.38	65.83	62.44	68.96	66.54	79.96	72.59	71.40	75.00
**word_tf**	**Acc**	68.94	80.17	69.70	71.18	73.35	63.60	72.61	63.70	63.64	65.38	65.96	73.88	66.02	66.98	69.24	67.87	81.15	70.63	72.91	74.33
	**F1-macro**	68.16	79.20	68.83	63.78	72.33	62.84	71.30	62.77	48.69	64.23	65.12	72.80	65.06	57.16	67.98	66.99	80.31	69.78	66.89	73.36
	**AUROC**	74.63	87.44	75.80	80.56	79.49	68.45	78.27	68.47	69.52	70.52	71.23	80.27	71.69	74.03	74.43	73.76	88.06	76.83	82.91	81.06
	**Precision-macro**	68.07	79.29	68.70	76.91	72.22	62.87	71.37	62.73	68.96	64.13	65.06	72.74	64.97	70.79	67.91	66.88	80.28	69.64	77.55	73.23
	**Recall-macro**	68.69	79.12	69.23	64.56	72.52	63.38	71.27	63.15	54.77	64.45	65.58	72.91	65.41	59.61	68.09	67.40	80.35	70.21	66.95	73.56
**word_tfidf**	**Acc**	68.80	79.90	69.85	70.75	73.35	63.73	72.31	63.47	63.88	65.08	66.15	74.19	66.09	67.85	69.33	67.86	80.53	70.48	72.66	74.37
	**F1-macro**	68.04	78.94	68.99	63.17	72.37	62.93	70.99	62.54	49.84	64.06	65.31	73.05	65.13	58.67	68.15	66.99	79.65	69.64	66.35	73.43
	**AUROC**	74.59	87.19	75.84	80.87	79.54	68.45	78.36	68.48	68.72	70.62	71.28	80.53	71.70	75.51	74.52	73.82	87.67	76.93	83.25	81.16
	**Precision-macro**	67.97	79.01	68.86	76.19	72.25	62.95	71.04	62.51	67.84	63.97	65.24	73.04	65.04	72.08	68.05	66.88	79.63	69.49	77.82	73.29
	**Recall-macro**	68.60	78.90	69.40	64.09	72.59	63.44	70.96	62.93	55.24	64.36	65.77	73.07	65.49	60.66	68.30	67.42	79.68	70.07	66.52	73.69
**LIWC**	**Acc**	56.65	57.40	58.16	60.69	57.84	56.28	56.73	57.49	60.48	57.25	55.84	56.74	57.30	60.47	57.24	56.44	57.73	58.27	60.54	58.27
	**F1-macro**	55.98	56.23	57.45	46.74	57.18	55.69	55.71	56.84	46.37	56.75	55.33	55.75	56.63	45.63	56.75	55.95	56.68	57.63	46.53	57.67
	**AUROC**	59.78	59.73	61.38	57.37	61.21	59.01	59.30	60.52	57.47	60.30	58.04	58.66	60.04	56.23	60.24	58.96	59.15	61.18	57.50	61.16
	**Precision-macro**	56.54	56.45	58.01	52.29	57.78	56.34	56.01	57.45	52.04	57.50	56.05	56.07	57.21	51.82	57.55	56.71	56.99	58.25	52.13	58.32
	**Recall-macro**	67.23	81.66	73.16	76.30	75.20	66.01	74.25	66.53	69.33	69.86	65.57	73.81	66.23	68.77	69.71	66.61	81.16	73.09	75.28	75.01

**Notes.**

* cnb = Complement Naïve Bayes, lgb = Light GBM, lgr = Logistic Regression, rf= Random Forest, svm = Support Vector Machine.

** combo is the combination of char_tf, char_tfidf, word_tf, word_tfidf and LIWC.

**Table A2:** Five-fold cross validation result average for Kaggle-Filtered dataset with MBTI keyword removed.

Feature	Dataset	Kaggle-Filtered					Kaggle-Filtered_noNN					Kaggle-Filtered_noNNnoSW					Kaggle-Filtered_noSW				
	Models	cnb	lgb	lgr	rf	svm	cnb	lgb	lgr	rf	svm	cnb	lgb	lgr	rf	svm	cnb	lgb	lgr	rf	svm
**combo**	**Acc**	61.22	66.26	59.28	62.01	64.04	59.66	61.47	56.66	61.09	60.95	59.59	61.83	57.99	60.81	60.61	60.68	65.89	60.65	61.90	63.78
	**F1-macro**	60.27	63.83	58.04	45.84	62.84	58.87	59.00	55.26	43.35	59.89	58.69	59.59	56.67	42.98	59.67	59.60	63.56	59.38	45.84	62.66
	**AUROC**	64.21	69.19	61.50	62.00	68.07	62.98	63.43	57.49	59.04	63.80	62.67	63.40	59.76	59.83	63.67	63.87	69.01	63.45	62.21	68.15
	**Precision-macro**	60.28	64.45	58.02	62.79	62.77	58.98	59.32	55.27	59.27	59.88	58.76	59.82	56.67	57.76	59.70	59.58	64.06	59.35	62.04	62.58
	**Recall-macro**	60.63	63.64	58.23	52.91	63.04	59.35	58.95	55.39	51.66	60.18	59.09	59.54	56.83	51.38	60.05	59.87	63.39	59.57	52.83	62.91
**char_tf**	**Acc**	60.02	63.77	60.38	61.16	62.57	58.70	61.44	57.77	60.63	60.10	58.69	61.20	57.69	60.62	60.09	59.53	64.21	60.66	61.43	62.10
	**F1-macro**	59.19	61.38	59.44	42.66	61.57	57.91	59.10	56.81	41.48	59.16	57.93	58.64	56.67	41.69	59.20	58.77	61.74	59.71	43.43	61.14
	**AUROC**	63.21	66.61	64.03	59.07	66.73	62.10	62.53	59.57	57.79	63.43	61.70	62.12	59.49	57.25	63.15	62.99	66.60	63.92	60.61	66.44
	**Precision-macro**	59.29	61.76	59.49	60.16	61.54	58.05	59.39	56.89	57.36	59.20	58.09	58.96	56.74	56.83	59.27	58.91	62.26	59.74	61.84	61.13
	**Recall-macro**	59.65	61.26	59.82	51.55	61.91	58.38	59.10	57.14	50.91	59.54	58.42	58.58	56.98	50.95	59.61	59.28	61.63	60.08	51.92	61.50
**char_tfidf**	**Acc**	59.82	64.50	60.50	61.36	62.31	58.63	61.26	57.24	60.92	59.81	58.60	61.17	57.32	60.47	59.65	59.55	63.83	60.94	61.43	62.24
	**F1-macro**	59.08	62.01	59.55	43.82	61.47	57.87	59.09	56.29	42.79	59.04	57.86	58.68	56.34	42.41	58.87	58.85	61.06	59.99	43.97	61.37
	**AUROC**	63.18	66.42	64.02	59.09	66.74	62.05	62.92	59.59	57.94	63.33	61.71	62.19	59.53	57.69	63.05	62.98	65.99	63.96	59.50	66.46
	**Precision-macro**	59.24	62.50	59.59	60.80	61.50	58.04	59.30	56.40	58.54	59.18	58.04	59.00	56.42	55.67	59.01	59.03	61.69	60.01	61.22	61.39
	**Recall-macro**	59.62	61.86	59.92	51.96	61.92	58.38	59.07	56.64	51.41	59.56	58.38	58.65	56.66	51.00	59.38	59.42	60.92	60.36	52.05	61.80
**word_tf**	**Acc**	61.13	65.80	60.30	61.22	61.76	59.25	60.03	56.32	60.29	59.62	60.24	60.65	58.69	60.99	60.70	62.30	65.52	61.21	62.12	63.88
	**F1-macro**	60.33	63.99	59.32	42.70	60.74	58.45	58.60	55.32	40.61	58.57	59.37	59.22	57.74	43.86	59.69	61.32	63.91	60.22	46.13	62.86
	**AUROC**	64.36	69.11	63.30	60.13	65.73	62.45	62.27	58.22	56.91	62.33	63.55	63.93	61.10	59.25	64.10	65.49	69.35	65.44	62.85	67.81
	**Precision-macro**	60.40	64.13	59.36	61.20	60.73	58.58	58.56	55.43	53.95	58.59	59.44	59.23	57.80	58.20	59.70	61.30	63.94	60.24	63.17	62.82
	**Recall-macro**	60.81	63.92	59.68	51.61	61.07	58.93	58.71	55.63	50.47	58.87	59.80	59.37	58.09	51.73	60.03	61.67	63.90	60.58	53.06	63.19
**word_tfidf**	**Acc**	60.95	64.85	60.02	61.20	61.63	59.23	59.64	56.30	60.76	59.31	60.33	60.76	58.64	61.16	60.79	62.31	65.27	61.25	62.31	63.75
	**F1-macro**	60.13	62.75	59.03	44.09	60.83	58.44	57.93	55.35	42.42	58.46	59.48	59.15	57.71	44.75	59.93	61.36	63.46	60.27	47.16	62.83
	**AUROC**	64.35	68.08	63.30	60.53	65.81	62.47	61.66	58.19	57.30	62.41	63.58	63.19	61.12	60.37	64.19	65.51	69.02	65.45	62.74	67.90
	**Precision-macro**	60.20	63.06	59.07	59.56	60.90	58.57	57.93	55.47	57.61	58.55	59.55	59.14	57.78	58.84	59.99	61.35	63.60	60.28	62.86	62.83
	**Recall-macro**	60.60	62.65	59.38	51.92	61.33	58.92	57.96	55.68	51.21	58.88	59.92	59.20	58.07	52.05	60.38	61.74	63.40	60.63	53.47	63.25
**LIWC**	**Acc**	56.58	57.08	58.19	60.65	58.02	56.45	57.59	57.65	59.77	57.42	55.55	56.94	57.57	60.29	57.76	56.15	58.24	58.28	60.55	57.95
	**F1-macro**	55.88	55.99	57.51	46.46	57.34	55.89	56.45	57.01	45.62	56.85	55.10	55.65	56.91	45.72	57.20	55.68	56.89	57.63	46.32	57.36
	**AUROC**	59.84	59.37	61.44	58.10	61.39	59.37	59.30	60.93	57.50	60.77	57.96	58.08	60.15	56.81	60.44	58.54	59.74	61.12	57.90	61.22
	**Precision-macro**	56.14	56.04	57.75	56.46	57.60	56.29	56.50	57.29	53.91	57.21	55.64	55.68	57.19	55.14	57.56	56.19	56.90	57.89	56.08	57.70
	**Recall-macro**	56.40	56.25	58.08	52.17	57.92	56.57	56.70	57.61	51.36	57.54	55.90	55.83	57.51	51.73	57.90	56.47	57.06	58.24	52.08	58.04

**Notes.**

* cnb = Complement Naïve Bayes, lgb = Light GBM, lgr = Logistic Regression, rf= Random Forest, svm = Support Vector Machine.

** combo is the combination of char_tf, char_tfidf, word_tf, word_tfidf and LIWC.

**Table A3:** Five-fold cross validation result standard deviation for Kaggle dataset without MBTI keyword removed.

Features	Dataset	Kaggle					Kaggle_noNN					Kaggle_noNNnoSW					Kaggle_noSW				
	Models	cnb	lgb	lgr	rf	svm	cnb	lgb	lgr	rf	svm	cnb	lgb	lgr	rf	svm	cnb	lgb	lgr	rf	svm
**combo**	**Acc**	0.68	1.09	1.41	0.99	0.96	1.52	1.14	0.59	1.19	0.82	1.31	1.28	0.75	0.62	0.80	1.02	0.88	0.76	1.35	0.89
	**F1-macro**	0.76	1.14	1.34	1.27	1.01	1.55	1.09	0.53	1.42	0.77	1.31	1.23	0.76	0.61	0.88	1.11	0.98	0.76	1.65	0.95
	**AUROC**	0.92	1.22	1.28	1.67	0.73	1.11	1.20	0.80	1.80	0.55	1.10	1.36	0.80	1.63	0.67	1.19	1.35	0.70	1.39	0.85
	**Precision-macro**	0.72	1.17	1.36	1.13	1.01	1.54	1.22	0.53	2.26	0.83	1.29	1.37	0.74	1.58	0.85	1.08	0.89	0.76	1.50	0.92
	**Recall-macro**	0.81	1.13	1.27	1.20	1.02	1.56	1.03	0.52	1.22	0.72	1.29	1.15	0.77	0.55	0.95	1.16	1.06	0.76	1.54	1.00
**char_tf**	**Acc**	0.80	0.96	1.65	1.43	1.52	1.11	1.25	1.53	1.35	1.47	1.29	1.34	0.92	0.88	1.40	0.54	0.80	0.93	0.76	1.36
	**F1-macro**	0.88	1.04	1.63	1.85	1.52	1.12	1.14	1.46	1.74	1.53	1.30	1.22	0.83	1.08	1.44	0.60	0.90	0.96	1.07	1.39
	**AUROC**	0.93	1.25	1.16	2.01	1.20	0.99	1.46	1.44	1.58	1.66	0.78	1.54	1.18	1.49	1.40	0.77	1.22	0.93	1.51	0.93
	**Precision-macro**	0.91	1.01	1.63	1.68	1.51	1.10	1.31	1.41	2.71	1.51	1.26	1.37	0.79	1.81	1.43	0.63	0.81	0.95	0.79	1.38
	**Recall-macro**	0.99	1.12	1.57	1.65	1.48	1.12	1.03	1.40	1.43	1.57	1.30	1.09	0.75	0.91	1.46	0.68	1.03	0.99	0.94	1.42
**char_tfidf**	**Acc**	0.81	1.36	1.58	0.89	1.34	1.24	1.00	1.59	1.26	1.47	1.18	1.47	1.27	1.31	1.36	0.83	1.07	1.27	0.66	1.17
	**F1-macro**	0.90	1.42	1.58	1.10	1.31	1.28	1.04	1.51	1.62	1.48	1.23	1.40	1.23	1.84	1.40	0.91	1.17	1.26	0.94	1.13
	**AUROC**	0.88	1.37	1.19	1.45	1.17	0.94	1.44	1.54	1.89	1.56	0.72	1.56	1.49	1.08	1.39	0.72	1.48	1.02	1.03	0.89
	**Precision-macro**	0.92	1.45	1.57	1.21	1.30	1.26	1.06	1.46	2.63	1.46	1.22	1.57	1.19	2.26	1.39	0.93	1.10	1.24	0.60	1.12
	**Recall-macro**	0.99	1.42	1.53	0.98	1.24	1.32	1.06	1.43	1.33	1.47	1.28	1.31	1.19	1.47	1.43	1.00	1.25	1.22	0.84	1.06
**word_tf**	**Acc**	0.35	1.04	1.21	0.90	0.92	1.31	0.83	0.85	0.46	1.18	1.65	1.09	1.74	0.96	1.47	0.85	2.00	1.12	1.30	1.47
	**F1-macro**	0.38	1.17	1.16	1.41	0.86	1.34	0.80	0.83	1.18	1.26	1.70	1.09	1.76	2.25	1.68	0.95	2.14	1.19	2.17	1.61
	**AUROC**	0.25	1.35	1.17	1.11	1.03	1.21	1.06	1.10	1.05	1.22	1.37	1.47	1.69	1.14	1.52	1.26	1.65	1.24	1.81	1.44
	**Precision-macro**	0.40	1.05	1.13	1.13	0.90	1.32	0.87	0.81	2.04	1.25	1.68	1.12	1.74	0.64	1.61	0.95	2.06	1.17	0.80	1.55
	**Recall-macro**	0.48	1.28	1.11	1.09	0.78	1.37	0.78	0.83	0.61	1.30	1.73	1.07	1.76	1.45	1.77	1.04	2.24	1.26	1.75	1.69
**word_tfidf**	**Acc**	0.31	1.30	1.40	1.43	0.95	1.37	0.75	1.23	0.82	1.25	1.69	1.61	1.82	0.70	1.43	0.91	1.55	1.24	0.94	1.53
	**F1-macro**	0.39	1.47	1.35	2.33	0.86	1.38	0.83	1.24	1.61	1.29	1.73	1.64	1.84	1.23	1.59	1.01	1.71	1.26	1.31	1.71
	**AUROC**	0.24	1.26	1.21	1.58	0.93	1.11	1.05	1.16	1.20	1.14	1.35	1.34	1.73	2.29	1.43	1.23	1.48	1.26	1.48	1.39
	**Precision-macro**	0.42	1.33	1.31	1.78	0.91	1.37	0.81	1.23	2.07	1.28	1.71	1.68	1.81	1.40	1.55	1.01	1.59	1.23	1.42	1.62
	**Recall-macro**	0.52	1.62	1.28	1.77	0.75	1.41	0.87	1.26	1.01	1.31	1.76	1.61	1.84	0.86	1.67	1.09	1.84	1.27	1.09	1.85
**LIWC**	**Acc**	1.66	1.15	1.13	0.79	1.06	1.37	1.10	0.63	0.96	0.86	1.48	1.22	1.17	0.41	0.49	0.76	1.14	0.84	0.67	1.66
	**F1-macro**	1.60	1.08	1.05	1.18	1.00	1.27	1.01	0.66	1.28	0.78	1.45	1.07	1.21	0.19	0.54	0.77	1.17	0.86	0.89	1.63
	**AUROC**	1.76	1.43	1.05	0.77	0.95	1.51	1.46	0.78	1.42	0.60	1.66	1.13	0.97	0.94	1.17	1.03	1.31	1.34	1.09	1.42
	**Precision-macro**	1.50	1.04	0.98	2.34	0.93	1.15	0.95	0.68	2.88	0.65	1.38	0.98	1.21	1.34	0.61	0.78	1.19	0.87	1.93	1.58
	**Recall-macro**	1.56	1.06	1.01	0.85	0.97	1.19	0.97	0.71	0.94	0.68	1.44	0.99	1.27	0.31	0.64	0.82	1.23	0.91	0.69	1.64

**Notes.**

* cnb = Complement Naïve Bayes, lgb = Light GBM, lgr = Logistic Regression, rf= Random Forest, svm = Support Vector Machine.

** combo is the combination of char_tf, char_tfidf, word_tf, word_tfidf and LIWC.

**Table A4:** Five-fold cross validation result standard deviation for Kaggle-Filtered dataset with MBTI keyword removed.

Feature	Dataset	Kaggle-Filtered					Kaggle-Filtered_noNN					Kaggle-Filtered_noNNnoSW					Kaggle-Filtered_noSW				
	Models	cnb	lgb	lgr	rf	svm	cnb	lgb	lgr	rf	svm	cnb	lgb	lgr	rf	svm	cnb	lgb	lgr	rf	svm
**combo**	**Acc**	1.00	0.99	1.42	0.35	0.83	1.00	0.78	1.10	0.17	0.85	0.89	1.26	0.97	0.72	0.93	1.38	1.16	0.91	0.28	0.80
	**F1-macro**	0.94	0.88	1.40	0.41	0.84	0.94	0.59	0.99	0.58	0.75	0.87	1.21	0.87	1.17	0.94	1.39	1.23	0.75	0.60	0.81
	**AUROC**	0.93	0.55	1.41	1.27	1.24	0.78	0.77	1.12	1.00	0.89	0.99	0.79	0.88	0.83	1.09	0.82	1.16	1.21	0.62	0.88
	**Precision-macro**	0.90	1.08	1.38	1.81	0.83	0.89	0.62	0.96	1.03	0.71	0.84	1.19	0.85	4.18	0.93	1.36	1.23	0.73	1.21	0.79
	**Recall-macro**	0.91	0.85	1.40	0.35	0.85	0.91	0.56	0.96	0.23	0.69	0.87	1.19	0.83	0.74	0.97	1.40	1.20	0.68	0.33	0.81
**char_tf**	**Acc**	0.85	0.96	1.79	0.61	1.04	0.83	0.80	0.92	0.48	0.58	1.04	0.92	1.11	0.28	0.66	1.12	0.75	1.70	0.40	0.75
	**F1-macro**	0.85	0.89	1.68	1.33	1.02	0.87	1.14	0.89	0.37	0.64	1.07	0.97	1.01	0.93	0.57	1.14	0.56	1.68	0.71	0.70
	**AUROC**	0.99	0.60	1.38	0.90	0.84	1.32	0.43	1.03	0.73	0.56	1.26	0.95	0.66	1.13	0.56	0.82	0.60	1.45	1.48	0.88
	**Precision-macro**	0.82	0.98	1.59	4.16	0.99	0.88	0.94	0.86	3.81	0.66	1.07	0.97	0.97	2.47	0.52	1.12	0.69	1.63	2.53	0.66
	**Recall-macro**	0.85	0.87	1.61	0.73	1.01	0.92	1.13	0.89	0.40	0.70	1.11	0.95	0.98	0.36	0.52	1.17	0.58	1.67	0.45	0.67
**char_tfidf**	**Acc**	1.15	0.98	1.63	0.64	0.69	1.11	1.11	1.20	0.53	0.85	1.08	0.73	0.74	0.64	0.71	1.16	0.87	1.72	0.47	0.78
	**F1-macro**	1.18	1.16	1.52	1.05	0.64	1.13	0.57	1.18	1.05	0.92	1.10	0.89	0.71	1.04	0.61	1.17	0.84	1.65	0.70	0.71
	**AUROC**	1.02	1.03	1.44	0.99	0.93	1.33	0.67	1.14	1.60	0.67	1.23	0.75	0.67	0.79	0.64	0.80	1.07	1.34	1.01	0.94
	**Precision-macro**	1.17	1.11	1.44	3.99	0.61	1.12	0.78	1.15	3.14	0.96	1.08	0.73	0.69	3.71	0.54	1.15	0.94	1.59	2.88	0.67
	**Recall-macro**	1.23	1.14	1.45	0.69	0.61	1.16	0.52	1.19	0.59	1.01	1.13	0.86	0.71	0.68	0.54	1.20	0.81	1.62	0.48	0.68
**word_tf**	**Acc**	0.72	0.73	1.49	0.42	0.83	0.53	0.66	0.73	0.40	1.17	1.04	1.19	0.48	0.25	0.64	0.56	1.74	0.60	0.33	0.80
	**F1-macro**	0.74	0.78	1.44	0.86	0.88	0.47	0.62	0.76	0.86	1.19	1.12	1.00	0.49	1.79	0.78	0.58	1.76	0.71	0.96	0.78
	**AUROC**	0.62	0.73	1.37	1.04	0.40	0.91	0.56	0.67	1.03	0.88	0.83	0.94	0.69	1.51	0.76	0.75	1.01	1.17	1.35	0.73
	**Precision-macro**	0.73	0.77	1.38	3.41	0.89	0.41	0.62	0.77	3.60	1.17	1.13	1.02	0.49	0.68	0.79	0.58	1.78	0.74	1.71	0.77
	**Recall-macro**	0.76	0.80	1.41	0.47	0.95	0.42	0.62	0.81	0.42	1.21	1.19	0.99	0.51	0.62	0.86	0.61	1.75	0.81	0.40	0.81
**word_tfidf**	**Acc**	0.61	1.16	1.59	0.57	0.82	0.61	0.86	0.81	0.39	0.68	1.01	1.60	0.45	0.54	0.74	0.56	1.24	0.85	0.37	0.81
	**F1-macro**	0.65	0.78	1.58	0.69	0.82	0.53	0.67	0.86	0.69	0.70	1.07	1.58	0.45	0.87	0.76	0.54	1.10	0.95	1.37	0.78
	**AUROC**	0.61	0.43	1.30	1.63	0.49	0.92	0.31	0.66	1.96	0.91	0.82	1.11	0.69	1.20	0.62	0.71	1.15	1.20	0.76	0.68
	**Precision-macro**	0.65	1.11	1.54	3.21	0.83	0.46	0.71	0.88	2.54	0.70	1.07	1.57	0.45	2.38	0.75	0.53	1.21	0.95	0.73	0.76
	**Recall-macro**	0.69	0.71	1.58	0.56	0.87	0.47	0.64	0.92	0.42	0.73	1.12	1.58	0.47	0.55	0.78	0.54	1.07	1.03	0.62	0.80
**LIWC**	**Acc**	1.50	1.29	1.47	0.62	1.28	1.25	1.34	1.30	0.76	0.89	1.02	1.37	0.90	0.51	0.86	0.52	1.14	0.81	0.09	0.92
	**F1-macro**	1.45	1.34	1.42	0.58	1.22	1.24	1.28	1.33	0.77	0.90	1.06	1.30	0.87	1.28	0.85	0.49	1.37	0.83	0.67	0.93
	**AUROC**	1.75	1.62	1.05	1.26	0.96	1.63	1.01	1.05	2.12	0.83	1.34	1.61	0.67	1.08	1.15	0.81	1.67	1.13	1.44	1.23
	**Precision-macro**	1.36	1.34	1.35	1.95	1.15	1.19	1.26	1.34	2.20	0.89	1.09	1.27	0.82	1.74	0.83	0.47	1.40	0.84	0.30	0.91
	**Recall-macro**	1.41	1.39	1.40	0.54	1.19	1.24	1.28	1.40	0.66	0.93	1.13	1.29	0.86	0.69	0.86	0.49	1.50	0.87	0.24	0.96

**Notes.**

* cnb = Complement Naïve Bayes, lgb = Light GBM, lgr = Logistic Regression, rf= Random Forest, svm = Support Vector Machine.

** combo is the combination of char_tf, char_tfidf, word_tf, word_tfidf and LIWC.

## Additional Information and Declarations

### Data Availability

The following information was supplied regarding data availability: The source code is available at GitHub: https://github.com/EnJunChoong/MBTI_JP_Prediction. The datasets are available at: Choong, EnJun (2021): 8k MBTI Dataset From Personality Cafe. figshare. Dataset. https://doi.org/10.6084/m9.figshare.14587572.v1
This data came from Kaggle: https://www.kaggle.com/datasnaek/mbti-type. The Twitter<sup>1<sup>dataset from Plank & Hovy, 2015 (Corpus of 1.2M English tweets (1,500 authors) annotated for gender and MBTI) is available at https://bitbucket.org/bplank/wassa2015/src/master/
The Twitter<sup>2<sup>dataset from Verhoeven, Plank & Daelemans (2016) (TwiSty is a corpus developed for research in author profiling. It contains personality (MBTI) and gender annotations for a total of 18,168 authors spanning six languages) is availble at: https://www.uantwerpen.be/en/research-groups/clips/research/datasets/
The Kaggle<sup>3<sup>dataset used in our research is available at Kaggle: https://www.kaggle.com/datasnaek/mbti-type/metadata. The Reddit<sup>4<sup>dataset (MBTI9k is a dataset of Reddit posts and comments labeled with MBTI personality types) is available upon request at: http://takelab.fer.hr/data/mbti.
